# Long-term survival with repeat resection for lung oligometastasis from pancreatic ductal adenocarcinoma: a case report

**DOI:** 10.1186/s40792-018-0435-2

**Published:** 2018-03-27

**Authors:** Ryota Matsuki, Masanori Sugiyama, Hidefumi Takei, Haruhiko Kondo, Masachika Fujiwara, Junji Shibahara, Junji Furuse

**Affiliations:** 1 Department of Surgery, School of Medicine, Kyorin University, 6-20-2 Shinkawa, Mitaka, Tokyo 181-8611 Japan; 2 Department of General Thoracic Surgery, School of Medicine, Kyorin University, Mitaka, Japan; 3 Department of Pathology, School of Medicine, Kyorin University, Mitaka, Japan; 4 Department of Medical Oncology, School of Medicine, Kyorin University, Mitaka, Japan

**Keywords:** Pancreatic ductal adenocarcinoma, Oligometastasis, Pulmonary metastasectomy

## Abstract

**Background:**

Long-term survival after resection of metastases from pancreatic ductal adenocarcinoma is rare.

**Case presentation:**

A 54-year-old man underwent pancreaticoduodenectomy (PD) for pancreatic ductal adenocarcinoma (PDAC) with UICC staging pT3N1M0 followed by adjuvant chemotherapy with gemcitabine (GEM). Three years after radical resection of the primary tumor, a tiny nodule was found in the lower lobe of the left lung. Despite treatment with GEM, it increased gradually, but no other metastases were found. Eighteen months after the first indication of the nodule, wedge resection was performed. Pathological examination of the nodule indicated a metastatic tumor from PDAC. Pulmonary metastasectomy was again performed for lung oligometastases at 77 and 101 months after PD. The patient has been asymptomatic without tumor recurrence for 4 years since the last pulmonary resection.

**Conclusions:**

In PDAC, the treatment strategy for oligometastasis is controversial. However, a few cases of long-term survival after pulmonary metastasectomy for oligometastasis of PDAC have been reported. More such cases need to be studied to address this issue effectively.

## Background

Surgical resection for metastases or recurrences of pancreatic ductal adenocarcinoma (PDAC) is not widely accepted because of frequent relapses with unlimited and aggressive growth. Systemic chemotherapy is commonly used for metastases or recurrences of PDAC. Recently, single cases and small series of surgical resection for oligometastasis from PDAC have been reported [1–4]. A case of long-term survival after three pulmonary metastasectomies for oligometastasis of PDAC is reported.

## Case presentation

**Fig. 1:**
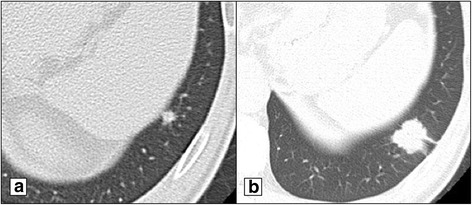
Imaging studies of the first pulmonary metastasis. Chest CT (53 months after curative resection of the primary pancreatic tumor) shows a tiny nodule at the lower lobe of the left lung (**a**). Follow-up CT (70 months after curative resection of the primary pancreatic tumor) shows that this nodule has enlarged to 2 cm in size with spiculation (**b**). It is difficult to determine whether this nodule is a primary or metastatic tumor on imaging

A 54-year-old man underwent pancreaticoduodenectomy (PD) for PDAC in our hospital in April 2004. Histopathological examination confirmed well-differentiated adenocarcinoma with UICC staging T3 (CH+, DU+, S+, RP+, PV−, A−, PL−, OO−) N1M0 stage IIB (UICC 7th edition). The diameter of the main pancreatic tumor was 40 mm in size. Number 13a lymph node was positive for metastasis. Resection margins of surgical specimen was all negative for caner (R0 resection). After surgical resection, adjuvant chemotherapy with gemcitabine (GEM) was performed until January 2006. Follow-up computed tomography (CT) and blood examination were performed every 6 months after primary resection. During the follow-up, serum carcinoembryonic antigen (CEA) level and carbohydrate antigen (CA) 19-9 level were within normal range [1.9–4.1 ng/mL (normal range, > 5.0) and 8.0–24.2 U/mL (normal range, > 37), respectively]. In April 2007, follow-up CT showed two tiny nodules at the lower lobe of the left lung and the lower lobe of the right lung (Fig. 1a). The serum CEA level and CA19-9 level were within normal range. However, there were no symptoms and evidence of inflammatory changes in the laboratory data during the follow-up [WBC 6500–7700/μL (normal 3300–8600) and CRP 0.1–1.7 mg/dL (normal 0–0.14)], it was difficult to differentiate these tiny nodules from inflammatory change to metastatic nodules. During the follow-up, the nodule in the lower lobe of the left lung gradually enlarged to a 2-cm nodule with spiculation in September 2008 (Fig. 1b). There was no change in the tiny nodule of the lower lobe of the right lung.

Percutaneous lung biopsy showed atypical cells in granulation tissue. Differentiation between a primary and metastatic tumor was difficult on preoperative imaging studies and the percutaneous lung biopsy specimen. Furthermore, no other tumors were found on imaging examinations. Therefore, wedge resection of the left lower lobe of the lung was performed in October 2008.

**Fig. 2:**
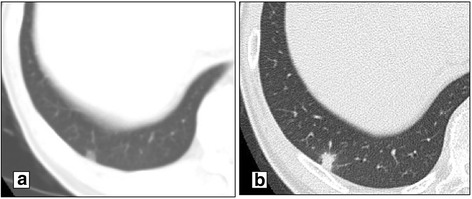
Imaging studies of the second pulmonary metastasis. Chest CT (at the same time when another nodule was found in the lower lobe of the left lung) shows a tiny nodule in the lower lobe of the right lung. It is difficult to determine whether this nodule is a metastatic tumor or an inflammatory nodule. **a** Follow-up chest CT (94 months after curative resection of the primary pancreatic tumor) shows that this nodule has increased gradually. **b** It is diagnosed as a pulmonary metastasis from PDAC

**Fig. 3:**
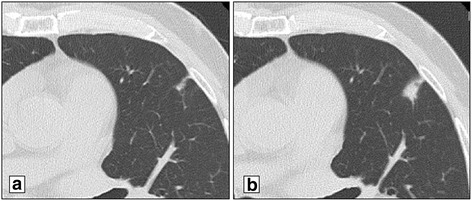
Imaging studies of the third pulmonary metastasis. Follow-up chest CT (98 months after curative resection of the primary pancreatic tumor) shows a newly appearing nodule in the upper lobe of the left lung (**a**). Chest CT (108 months after curative resection of the primary pancreatic tumor) shows that this nodule has increased (**b**) and appears to be a pulmonary metastasis from PDAC

Histopathological examination showed that the tumor was composed of columnar cancer cells with papillotubular proliferation. These findings were similar to those of the primary pancreatic cancer and indicated a pulmonary metastasis (PM) from PDAC. After surgery, adjuvant chemotherapy with GEM was completed for 6 months. Follow-up CT and blood examination were performed every 3 months after pulmonary metastasectomy. However, the nodule in the lower lobe of the right lung, which was detected in April 2007, gradually enlarged from March 2010, about 1 year and 10 months later from the completion of adjuvant chemotherapy (Fig. 2a,b,). During follow-up, no other tumors appeared, and wedge resection of the lower lobe of the right lung was performed in September 2010. Histopathological examination also confirmed PM from PDAC. In November 2011, a new, enlarging, solitary nodule was found in the left upper lobe of the left lung (Fig. 3). No other metastatic tumors were observed on imaging studies including fluorodeoxyglucose-positron emission tomography (FDG-PET). Thus, pulmonary metastasectomy was performed for the third time in September 2012. Histopathological examination also confirmed PM from PDAC. Eventually, adjuvant chemotherapy with GEM was performed from December 2008 (after the initial pulmonary metastasectomy) to May 2013 (8 months after the last pulmonary metastasectomy). Adjuvant chemotherapy with GEM was completed for 6 months again. The patient has been asymptomatic without tumor recurrence or elevated tumor markers for 4 years since the last pulmonary metastasectomy.

## Discussion

This was a rare case that underwent pulmonary metastasectomy for recurrence of PDAC three times and achieved long-term relapse-free survival. Surgical resection of metastatic tumors or recurrent tumors of PDAC has not become an established therapy because of frequent relapses with unlimited growth and rapid progression. Recently, a few cases that showed the effectiveness of metastasectomy for oligometastasis of PDAC have been reported [1–4].

**Fig. 4:**
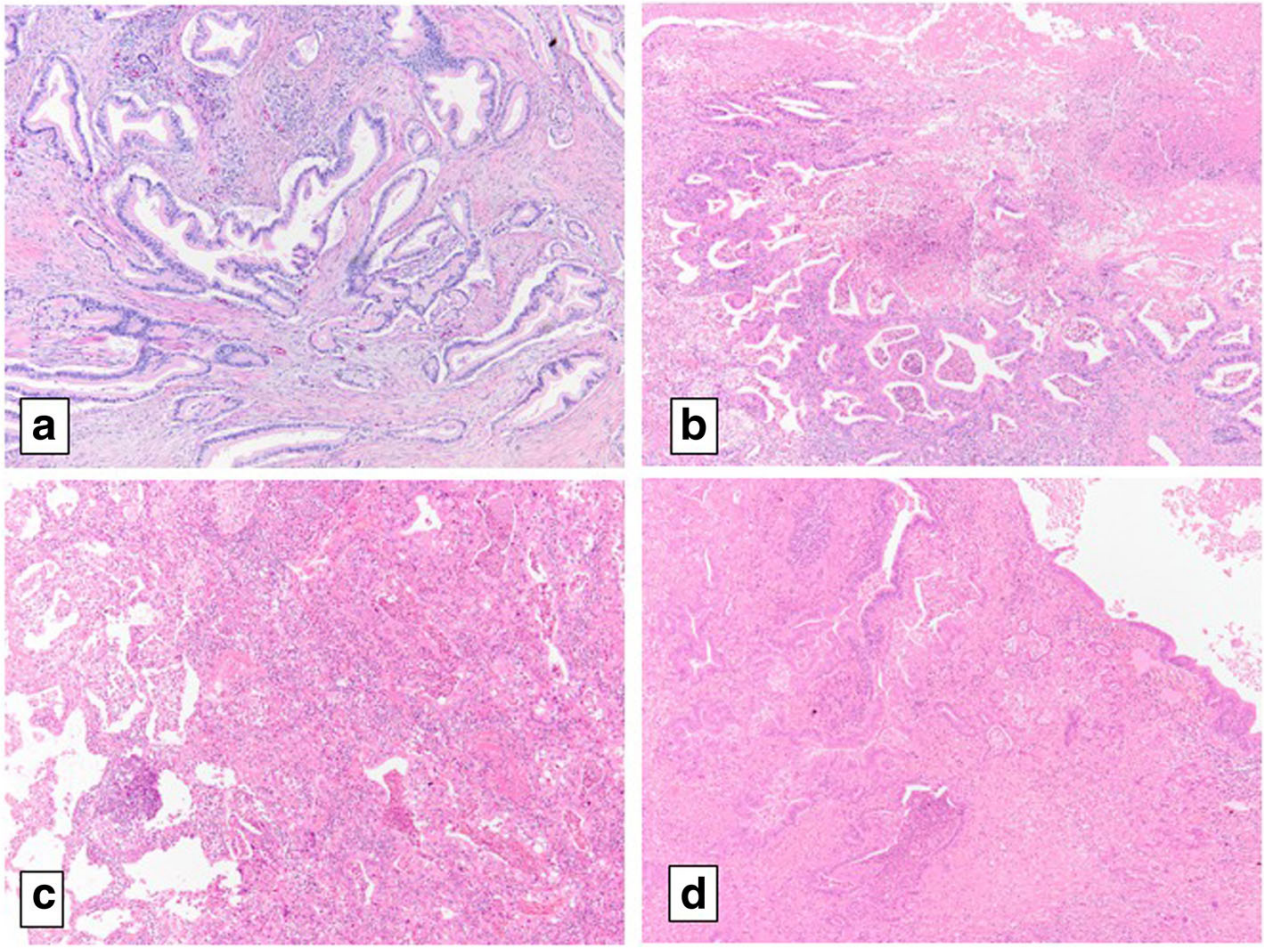
Histopathology. **a** Microscopic view of the primary tumor. **b** Microscopic view of the first pulmonary metastatic tumor. **c** Microscopic view of the second pulmonary metastatic tumor. **d** Microscopic view of the third pulmonary metastatic tumor. Histologically, all of the metastatic tumors are composed of columnar cancer cells with papillotubular proliferation. These findings are similar to those of the primary tumor

Isolated pulmonary metastases as the first site of recurrence after PDAC resection represent a rare subgroup. Previous studies reported that the rates of an isolated lung recurrence were 3 and 22% among the first site of tumor recurrences resected after PDAC resection [4–9]. Recently, it has been reported that patients with pulmonary metastasis after PDAC resection have a better prognosis than patients with another site of metastasis [7, 9–11] (Fig. 4). A pattern of lung metastasis as the first recurrence may be a favorable prognostic factor in patients with relapsed PDAC; however, the mechanism remained unclear. Zheng et al. reported that patients with lung metastasis as the primary recurrence had lower pT category and less vascular invasion than patients with other metastases; it may relate to the prognosis [11].

In our case, peripancreatic infiltration was found but vascular invasion was not found.

Pulmonary metastasectomy has evolved for other cancers with synchronous or metachronous metastatic disease, such as colorectal adenocarcinoma, with a demonstrated survival benefit [12–15]. In PDAC, Arnaoutakis et al. reported that, in patients with isolated PM from PDAC, median cumulative survival was significantly improved in the pulmonary metastasectomy group compared with the chemotherapy group (51 vs 23 months); they considered that a relatively long interval between the initial PDAC resection and tumor relapse, with isolated and stable disease over time, and a favorable response to systemic chemotherapy indicated “good biology” [4]. Thomas et al. reported a survival benefit in patients who had disease-free survival prior to recurrence > 20 months, and the greatest benefit was seen in patients with isolated pulmonary metastases [16]. The previously reported small series showed that the median interval between the initial resection and pulmonary metastasectomy was 29.3–48 months and the overall survival after pulmonary metastasectomy was 18.6–38 months respectively [4, 17, 18]. However, to the best of our knowledge, no cases in which pulmonary metastasectomy was performed three times have been reported in the English literature.

In this case, adjuvant chemotherapy using GEM was performed after PM according to the primary resection. The benefit of adjuvant chemotherapy cannot be determined in this case because of the re-recurrence of lung metastases after chemotherapy, with no other recurrence being found. In PDAC, the treatment strategy for oligometastasis has not been established. A few cases of long-term survival after pulmonary metastasectomy for oligometastasis of PDAC have been reported. It is controversial whether or not performing repeat metastasectomy from PDAC is of oncological benefit. As in this case, repeat metastasectomy may achieve long-term survival after primary resection of the PDAC; the present case is considered to have relatively “good biology” and be a potentially good candidate for metastasectomy of recurrence. Studies of more such cases are needed to address this issue.

## Conclusions

In PDAC, the treatment strategy for oligometastasis is controversial. However, a few cases of long-term survival after pulmonary metastasectomy for oligometastasis of PDAC have been reported. Metastasectomy may achieved long-term survival after primary resection of the PDAC Studies of more such cases are needed to address this issue.

## Abbreviations

CT: Computed tomography
FDG-PET: Fluorodeoxyglucose-positron emission tomography
GEM: Gemcitabine
PD: Pancreaticoduodenectomy
PDAC: Pancreatic ductal adenocarcinoma
PM: Pulmonary metastasis

## References

[1] Kleeff J, Reiser C, Hinz U, Bachmann J, Debus J, Jaeger D, Friess H, Büchler MW (2007). Surgery for recurrent pancreatic ductal adenocarcinoma. Ann Surg.

[2] Tachezy M, Gebauer F, Janot M, Uhl W, Zerbi A, Montorsi M, Perinel J, Adham M, Dervenis C, Agalianos C, Malleo G, Maggino L, Stein A, Izbicki JR, Bockhorn M (2016). Synchronous resections of hepatic oligometastatic pancreatic cancer: disputing a principle in a time of safe pancreatic operations in a retrospective multicenter analysis. Surgery.

[3] Hackert T, Niesen W, Hinz U, Tjaden C, Strobel O, Ulrich A, Michalski CW, Büchler MW (2017). Radical surgery of oligometastatic pancreatic cancer. Eur J Surg Oncol.

[4] Arnaoutakis GJ, Rangachari D, Laheru DA, Iacobuzio-Donahue CA, Hruban RH, Herman JM, Edil BH, Pawlik TM, Schulick RD, Cameron JL, Meneshian A, Yang SC, Wolfgang CL (2011). Pulmonary resection for isolated pancreatic adenocarcinoma metastasis: an analysis of outcomes and survival. J Gastrointest Surg.

[5] Wangjam T, Zhang Z, Zhou XC, Lyer L, Faisal F, Soares KC, Fishman E, Hruban RH, Herman JM, Laheru D, Weiss M, Li M, De Jesus-Acosta A, Wolfgang CL, Zheng L (2015). Resected pancreatic ductal adenocarcinomas with recurrence limited in lung have a significantly better prognosis than those with other recurrence patterns. Oncotarget.

[6] Tzung-Kai CK, Singla S, Papavasiliou P, Arrangoiz R, Gaughan JP, Hoffman JP (2013). Patterns of recurrence and outcomes in pancreatic cancer. Gastrointestinal cancers symposium 2013.

[7] Katz MH, Wang H, Fleming JB, Sun CC, Hwang RF, Wolff RA, Varadhachary G, Abbruzzese JL, Crane CH, Krishnan S, Vauthey JN, Abdalla EK, Lee JE, Pisters PW, Evans DB (2009). Long-term survival after multidisciplinary management of resected pancreatic adenocarcinoma. Ann Surg Oncol.

[8] Yamashita K, Miyamoto A, Hama N, Asaoka T, Maeda S, Omiya H, Takami K, Doki Y, Mori M, Nakamori S (2015). Survival impact of pulmonary metastasis as recurrence of pancreatic ductal adenocarcinoma. Dig Surg.

[9] Kruger S, Haas M, Burger PJ, Ormanns S, Modest DP, Westphalen CB, Michl M, Kleespies A, Angele MK, Hartwig W, Bruns CJ, Niyazi M, Roeder F, Kirchner T, Werner J, Heinemann V, Boeck S (2016). Isolated pulmonary metastases define a favorable subgroup in metastatic pancreatic cancer. Pancreatology.

[10] Lovecek M, Skalicky P, Chudacek J, Szkorupa M, Svebisova H, Lemstrova R, Ehrmann J, Melichar B, Yogeswara T, Klos D, Vrba R, Havlik R, Mohelnikova-Duchonova B (2017). Different clinical presentations of metachronous pulmonary metastases after resection of pancreatic ductal adenocarcinoma: retrospective study and review of the literature. World J Gastroenterol.

[11] Zheng B, Ohuchida K, Yan Z, Okumura T, Ohtsuka T, Nakamura M (2017). Primary recurrence in the lung is related to favorable prognosis in patients with pancreatic cancer and postoperative recurrence. World J Surg.

[12] Goya T, Miyazawa N, Kondo H, Tsuchiya R, Naruke T, Suemasu K (1989). Surgical resection of pulmonary metastases from colorectal cancer. 10-year follow-up. Cancer.

[13] Onaitis MW, Petersen RP, Haney JC, Saltz L, Park B, Flores R, Rizk N, Bains MS, Dycoco J, D'Amico TA, Harpole DH, Kemeny N, Rusch VW, Downey R (2009). Prognostic factors for recurrence after pulmonary resection of colorectal cancer metastases. Ann Thorac Surg.

[14] Watanabe K, Nagai K, Kobayashi A, Sugito M, Saito N (2009). Factors influencing survival after complete resection of pulmonary metastases from colorectal cancer. Br J Surg.

[15] Okumura T, Boku N, Hishida T, Ohde Y, Sakao Y, Yoshiya K, Higashiyama M, Hyodo I, Mori K, Kondo H (2017). Surgical outcome and prognostic stratification for pulmonary metastasis from colorectal cancer. Ann Thorac Surg.

[16] Thomas RM, Truty MJ, Nogueras-Gonzalez GM, Fleming JB, Vauthey JN, Pisters PW, Lee JE, Rice DC, Hofstetter WL, Wolff RA, Varadhachary GR, Wang H, Katz MH (2012). Selective reoperation for locally recurrent or metastatic pancreatic ductal adenocarcinoma following primary pancreatic resection. J Gastrointest Surg.

[17] Boone BA, Zeh HJ, Mock BK, Johnson PJ, Dvorchik I, Lee K, Moser AJ, Bartlett DL, Marsh JW (2014). Resection of isolated local and metastatic recurrence in periampullary adenocarcinoma. HPB (Oxford).

[18] Tagawa T, Ito K, Fukuzawa K, Okamoto T, Yoshimura A, Kawasaki T, Masuda T, Iwaki K, Terashi T, Okamoto M, Shiromizu A, Motohiro A, Maehara Y (2017). Surgical resection for pulmonary metastasis from pancreatic and biliary tract cancer. Anticancer Res.

